# Exhaustive Effects of Dental Practice

**Published:** 1876-04

**Authors:** 


### EDITORIAL, ETC.

*Exhaustive Effects of Dental Practice.*—In a paper on this subject, read by John Allen before the American Dental Association, he contends that there is no profession or calling more arduous, or that taxes the mind and nervous system more severely than a heavy dental practice; and that the toil is often rendered doubly irksome by personal contact with patients whose nervous temperaments exhaust the vital force of the dental practitioner at a rapid rate. He gives the following melancholy record of those who died insane, as presenting a sad commentary in connection with this subject: Drs. L. Parmly, Weir, Wells, Snow, Flagg, Barrett, Blakesly, Griffin, Westcott, Franklin and Whitney, and says that to this list might be added many others of those who have lived and died in our own day. And in view of the facts presented, Dr. Allen thinks a spirit of inquiry should be aroused in our profession with reference to the cause of this peculiar fatality among our members. First, is it owing to the exhaustive nature of our calling? Or second, is it due to a course of habits, such as an excessive use of narcotics or stimulants, which an overworked brain cannot withstand? Or third, to a want of daily exercise in the open air? He recommends that this matter be brought before State and local societies with a view of eliciting further information upon this subject, and in order to bring the question in a tangible form before the Association, he offered the following resolution :

"*Resolved,* That a committee of three be appointed whose duty it shall be to collect facts with reference to the subject matter here presented, and also the best means of preserving the physical and mental capacities of dental practitioners," which was done.

